# Transcriptome Analysis of Hypothalamic Gene Expression during Daily Torpor in Djungarian Hamsters (*Phodopus sungorus*)

**DOI:** 10.3389/fnins.2017.00122

**Published:** 2017-03-13

**Authors:** Ceyda Cubuk, Julia Kemmling, Andrej Fabrizius, Annika Herwig

**Affiliations:** Zoologisches Institut, Universität HamburgHamburg, Germany

**Keywords:** metabolic depression, seasonal adaptations, circadian, hypothalamus, Illumina, RNA-Seq

## Abstract

Animals living at high or temperate latitudes are challenged by extensive changes in environmental conditions over seasons. Djungarian hamsters (*Phodopus sungorus*) are able to cope with extremely cold ambient temperatures and food scarcity in winter by expressing spontaneous daily torpor. Daily torpor is a circadian controlled voluntary reduction of metabolism that can reduce energy expenditure by up to 65% when used frequently. In the past decades it has become more and more apparent, that the hypothalamus is likely to play a key role in regulating induction and maintenance of daily torpor, but the molecular signals, which lead to the initiation of daily torpor, are still unknown. Here we present the first transcriptomic study of hypothalamic gene expression patterns in Djungarian hamsters during torpor entrance. Based on Illumina sequencing we were able to identify a total number of 284 differentially expressed genes, whereby 181 genes were up- and 103 genes down regulated during torpor entrance. The 20 most up regulated group contained eight genes coding for structure proteins, including five collagen genes, *dnha2* and *myo15a*, as well as the procoagulation factor *vwf*. In a proximate approach we investigated these genes by quantitative real-time PCR (qPCR) analysis over the circadian cycle in torpid and normothermic animals at times of torpor entrance, mid torpor, arousal and post-torpor. These qPCR data confirmed up regulation of *dnah2, myo15a*, and *vwf* during torpor entrance, but a decreased mRNA level for all other investigated time points. This suggests that gene expression of structure genes as well as the procoagulation factor are specifically initiated during the early state of torpor and provides evidence for protective molecular adaptions in the hypothalamus of Djungarian hamsters including changes in structure, transport of biomolecules and coagulation.

## Introduction

Metabolic depression (torpor) is a commonly used strategy of mammals to survive winter. A reduction in energy expenditure as well as energy requirements is necessary to compensate for harsh environmental conditions during winter season when ambient temperature (T_a_) drops and food availability is reduced (Jastroch et al., 2016).

The Djungarian hamster (also known as Siberian hamster, *P. sungorus*) has evolved a number of physiological and morphological adaptations (e.g., voluntary reduction of body weight, molt to dense white winter coat, gonadal regression, torpor) to seasonally reduce energy requirements (Figala et al., 1973; Scherbarth and Steinlechner, 2010). In Djungarian hamsters, winter adaptations are driven by photoperiod and can easily be induced by changes of the artificial light-dark cycle at moderate T_a_ in the laboratory (Steinlechner and Heldmaier, 1982; Vitale et al., 1985). The most effective adaptive trait is the expression of daily torpor that spontaneously occurs after 10–12 weeks in short days once all other physiological adaptations are completed and the corresponding hormonal systems are in winter state (reduced levels of prolactin, testosterone and leptin) (Cubuk et al., 2016). Daily torpor is initiated by an active depression of metabolic rate (25% below the level of resting metabolic rate), accompanied by reduced heart rate and ventilation as well as a decrease in body temperature (T_b_) to > 15°C and reduced physical locomotor activity (Heldmaier and Ruf, 1992; Heldmaier et al., 2004). Torpor bouts are usually timed into the light phase of the light-dark cycle and have been shown to be under circadian control. The average duration of a torpor episode is 6 h and it is terminated by a spontaneous arousal prior to the hamsters' naturally active phase (Kirsch et al., 1991). The incidence of daily torpor is highly variable between individuals as well as in the same animal (1–7 torpor bouts per week) and can save up to 65% of total energy requirements, when torpor is used on a daily basis (Heldmaier, 1981; Kirsch et al., 1991; Ruf et al., 1991).

Spontaneous daily torpor is dependent on signaling of various hormonal systems changing with seasons and morphology, nutritional state as well as circadian timing mechanisms, hence, the hypothalamus is the brain area most likely involved in its regulation. Manipulations of prolactin levels lead to reduced torpor incidence and when testosterone, leptin or T3 are supplemented peripherally torpor is almost completely blocked (Ouarour et al., 1991; Ruby et al., 1993; Freeman et al., 2004; Bank et al., 2015). It has already been shown, that lesion of various hypothalamic nuclei (suprachiasmatic nuclei, arcuate nucleus, paraventricular nucleus) alters torpor behavior. Moreover, the pharmacological activation of NPY mechanisms in arcuate nucleus induces torpor like hypothermia and hypothalamic application of T3 is able to block the expression of torpor (Ruby and Zucker, 1992; Ruby, 1995; Paul et al., 2005; Pelz and Dark, 2007; Dark and Pelz, 2008; Pelz et al., 2008). However, although torpor physiology has been extensively studied in this species, the regulatory systems in the brain ultimately initiating entrance into torpor on some days but not on others are entirely unknown.

Here we carried out a next generation sequencing (NGS) study to impartially screen for potential candidate genes playing a role in molecular hypothalamic torpor induction mechanisms. NGS allows the investigation of all transcripts of a genome by counting the number of mRNA sequencing reads of a specific tissue. To date, only few transcriptomic studies are available investigating gene expression patterns in the 13-lined ground squirrel (*Ictidomys tridecemlieatus*) during hibernation in various tissues, like cerebral cortex, hypothalamus, heart, skeletal muscle, brown adipose tissue and white adipose tissue (Hampton et al., 2011, 2013; Schwartz et al., 2013, 2015; Grabek et al., 2015). Except for one study investigating brown adipose tissue during entrance into torpor (Grabek et al., 2015), these studies were focused on time points before animals enter hibernation season, while being in deep hibernation or during the interbout arousals. The Djungarian hamster is an excellent animal model to investigate gene expression patterns during torpor entrance, because torpor is precisely timed into the circadian cycle and allows precise sampling with timed controls that are winter adapted but do not show torpor on that particular day. Moreover, substantial knowledge exists about hypothalamic mechanisms regulating seasonal adaptations in body weight and reproduction in this species (Ebling and Barrett, 2008).

Here we present a summary of differentially expressed genes during torpor entrance in the hypothalamus of *P. sungorus*. Moreover, we provide information about circadian regulation of mRNA expression patterns for selected candidate genes by relative gene expression analysis in torpid and normothermic hamsters.

## Materials and methods

### Animals

All experiments and procedures were conducted in accordance with the German Animal Welfare Law and approved by the local animal welfare authorities (No. 114_14, Hamburg, Germany). All animals originated from our own breeding colony at the Institute of Zoology University of Hamburg. Djungarian hamsters (*P. sungorus*) were bred and raised under artificial long photoperiod (16:8-h light:dark cycle) at 21°C ± 1°C T_a_. The animals were individually housed in plastic cages (Macrolon Type III). Before and during the experiments, hamsters were fed a hamster breeding diet (Altromin 7014, Germany) *ad libitum* and had free access to drinking water. For the experiments, 3–4 months old Djungarian hamsters were transferred to short day conditions (8:16-h light:dark cycle) at constant T_a_ of 18°C ± 1°C. After 12 weeks in short days they were implanted i.p. with DSI-transmitters (Model TA-F10, St. Paul, MN, USA) under 1.5–2% isoflurane anesthesia and carprofen (5 mg/kg) analgesia as previously described (Bank et al., 2015) to monitor T_b_ on line. T_b_ was recorded every 3 min.

### Experiment 1: transcriptomic analysis of hypothalamic gene expression at torpor entrance

#### Sampling

**Figure 1:**
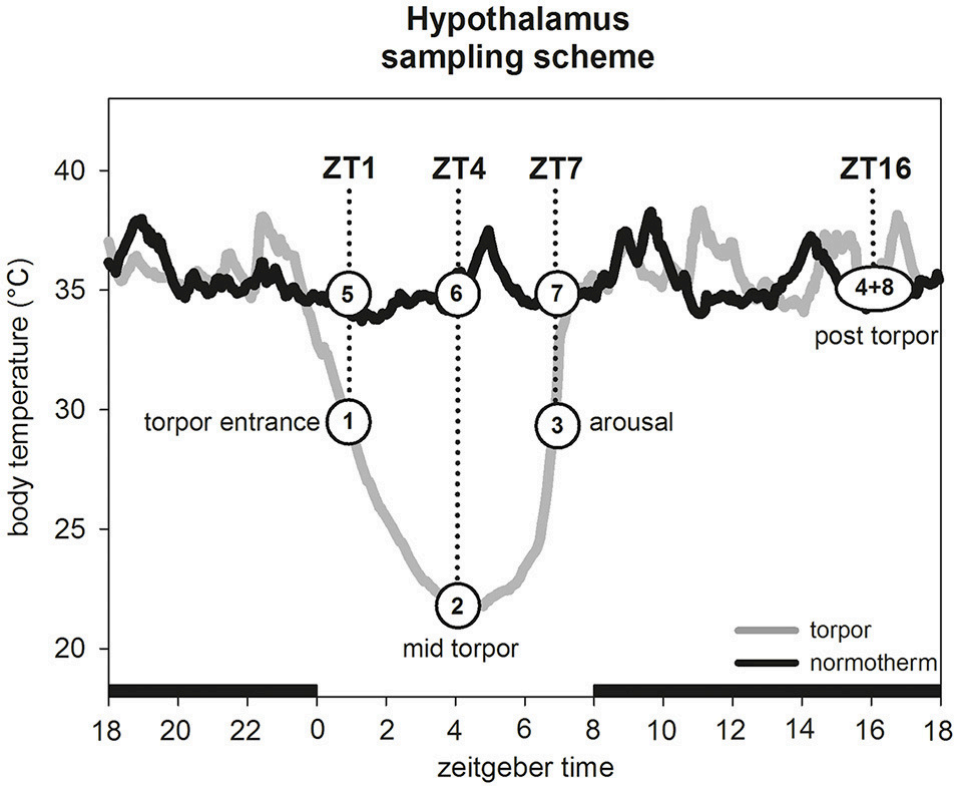
**Hypothalamus sampling scheme**. For experiment 1 one hamster group (*n* = 3) was sampled during torpor entrance (1) and a corresponding normothermic group (*n* = 3) at same ZT (5). For experiment 2 four hamster groups (*n* = 5 for each group) were sampled in the torpid state during torpor entrance at ZT1 (1), mid torpor at ZT4 (2), arousal at ZT7 (3) and post-torpor at ZT16 (4). For each time point a corresponding normothermic group (*n* = 5 for each group) was sampled (5–8).

Between week 13 and week 16, three animals were euthanized by carbon dioxide during entrance into torpor (T_b_ 30.4°C ± 0.6°C) at Zeitgebertime 1 (ZT1; ZT0 = lights on) (Figure 1, group 1). Additionally, three hamsters were culled in a normothermic state (T_b_ 36.1°C ± 0.7°C) at the same ZT as control group (Figure 1, group 5). The brain was dissected from each hamster, frozen on dry ice and stored at −80°C.

#### Isolation of total RNA

Hypothalamic blocks were cut from frozen tissues between Bregma −0.20 and −2.70 mm, laterally at the hypothalamic sulci and dorsally 3–4 mm from the ventral surface. Tissue samples were homogenized in 500 μl TriFast using a micropestle. Total RNA was obtained using peqGOLD Trifast™ (Peqlab, Erlangen, Germany) according to the manufacturer's instructions. Total RNA was purified with the Crystal RNA MiniKit (Biolabproducts, Bebensee, Germany) including an on-column digestion with RNase-free DNase (Qiagen, Hilden, Germany). RNA integrity was proven by gel electrophoresis, total RNA was quantified spectrometrically and RNA purity was assessed by the 260/280 nm ratio on a NanoDrop 1000 spectrophotometer.

#### Illumina sequencing

In total, 2 μg total RNA per sample were used for transcriptome analysis. Library preparation and Illumina sequencing were performed by *GENterprise* Genomics (Mainz, Germany). For library preparation the TruSeq RNA Library Preparation Kit (Illumina, San Diego, CA) was used. All RNA samples had a RIN ≥ 6.9. The samples were sequenced by Illumina NextSeq 500 with a calculated output of 50 million paired-end reads (2 × 150 bp) per sample. The raw Illumina data are available at the NCBI SRA database under the accession numbers biosample: SAMN062002211 to SAMN06200226 (Bioproject PRJNA360070). Since currently no annotated *Phodopus sungorus* genome is available, the reads were mapped against the genome of the Chinese hamster (*Cricetulus griseus*), that showed best compliance, using the CLC-Genomics Workbench 7.5.1 (Qiagen, Hilden, Germany). Only reads with intact pairs mapping with an 85% read identity and 85% read length were used for RPKM (reads per kilobase per million mapped reads) calculation (Mortazavi et al., 2008). Supplementary Table 1 shows the total number of reads and the number of reads mapped in pairs for each sample. Statistically significant expression changes between normothermic and torpid hamsters were calculated by an empirical analysis of digital gene expression (DGE) statistics, performing an “Exact Test” (Robinson and Smyth, 2008). This tool is implemented in CLC-Genomics Workbench 7.5.1. To correct for multiple testing, a false discovery rate (FDR) correction of *p*-values was applied (see Supplementary Table 2).

Transcripts with an RPKM-value ≥ 0.1 in one of the samples were chosen for further analysis. Transcripts with a fold change ≥ 1.2 and an FDR-corrected *p* ≤ 0.05 were considered as differentially expressed. The identified differentially expressed transcripts were functionally classified using the PANTHER Classification System (Protein ANalysis THrough Evolutionary Relationships; www.pantherdb.org) version 10.0 (Mi et al., 2013). Differentially expressed transcripts were additionally analyzed using the PANTHER Overrepresentation Test (release 20160715) applying the PANTHER GO-slim terms as annotated, followed by Bonferroni correction for multiple testing. The PANTHER Overrepresentation Test was conducted for all 284 differentially expressed genes as well as the 181 up regulated genes and the 103 down regulated genes respectively. *Mus musculus* was selected as reference organism for the GO annotation and for the statistical calculation of overrepresented GO-terms. Overrepresented terms with a Bonferroni corrected *p* ≤ 0.05 were considered as significant.

### Experiment 2: relative quantification of hypothalamic gene expression in different torpor stages

#### Sampling

To validate our Illumina results, we selected eight genes for further investigations by qPCR analysis. A group of seven genes immediately attracted attention for their potential role in structural changes (five collagens, myosin and dynein). Additionally, the von Willebrand factor (*vwf*) was chosen for its role in blood clotting. To provide more detailed information about gene expression changes over a circadian cycle in torpid and normothermic state, 40 hamsters were used for gene expression analysis by real-time PCR (qPCR). A total of 20 animals were culled by carbon dioxide on a day with torpor expression at ZT1 (entrance into torpor: T_b_ 30.8°C ± 0.5°C, *n* = 5), ZT4 (mid torpor: T_b_ 22.5°C ± 1.5°C, *n* = 5), ZT7 (arousal: T_b_ 30.4°C ± 0.4°C, *n* = 5) and ZT16 (active phase after torpor bout: T_b_ 35.7°C ± 0.6°C, *n* = 5) (Figure 1, groups 1–4). Five normothermic animals were sampled for each time point as respective control group (ZT1: Tb 35.7°C ± 0.5°C; ZT4: T_b_ 35.7°C ± 0.4°C; ZT7: T_b_ 35.6°C ± 0.4°C; ZT16: T_b_ 36.2°C ± 1.3°C) (Figure 1, groups 5–8). Brains were dissected, frozen on dry ice and stored at −80°C for qPCR analysis.

#### Isolation of RNA and cDNA synthesis

Hypothalami were dissected from frozen brains and isolation of total RNA was performed as described for Experiment 1. For qPCR templates and generation of standard plasmids, cDNA was synthesized from every total RNA sample using RevertAid H Minus First Strand cDNA Synthesis Kit (Thermo Scientific, Waltham, MA, USA) and oligo-(dT)18 oligonucleotide primers following manufacturer's instructions. Reverse transcription was conducted using 1 μg total RNA per sample.

#### Cloning and sequencing

For standard plasmids, coding sequence fragments (100–200 bp long) of collagen alpha-1(XXIV) chain-like (*LOC103164493*), collagen, type XX, alpha 1 (*col20a1*), collagen, type XVII, alpha 1 (*col17a1*), collagen, type XVIII, alpha 1 (*col18a1*), collagen, type V, alpha 3 (*col5a3*), dynein, axonemal, heavy chain 2 (*dnah2*), myosin XVA (*myo15a*), von Willebrand factor (*vwf*) and hypoxanthine phosphoribosyltransferase (*hprt*) were amplified by gene specific primers (Table 1). All primers were designed on the *P. sungorus* specific sequences generated by Illumina sequencing using the onlinetool OligoAnalyzer 3.1. The primers were designed with a melting temperature at 60°C ± 1.1°C. After cloning of the amplicons using the pGEM®-T Easy Vector System (Promega, Madison, USA) following the manufacturer's instructions, the cDNA fragments were Sanger-sequenced by GATC Biotech (Konstanz, Germany).

**Table 1:** ***P. sungorus* specific primer sequences**.

**Gene**		**5′3′sequence**	**Melting temperature (°C)**	**Amplicon length (bp)**
*LOC103164493*	Forward	CATGCAGCAGTAACGCCAACC	59.4	136
	Reverse	GTGGCAATTGTGCTTCACCAACTC	59.2	
*col20a1*	Forward	GCTCCTACCTCCACGTCTGTCTC	60.5	174
	Reverse	CTGCCATAGGTGTCACCTGCAC	60.2	
*col17a1*	Forward	CATAACCTCCTCCTGGGCTGATG	59.2	126
	Reverse	GCTCTTCCTACAGTGCTCCCATG	59.4	
*col18a1*	Forward	CAGGACCAAAGGGTGACAAAGGAG	59.7	189
	Reverse	GGCCAGGTACACTTGAGCTGAAG	59.8	
*col5a3*	Forward	GAACAAGGAGACCTCAAGGCTGAG	59.2	166
	Reverse	CTGCAAGACAGTGGCATTTCGTTC	58.9	
*dnah2*	Forward	CTTCGTGCTCAATGATATGGGCCG	60.6	102
	Reverse	CTGCGATGGCTCTTGTCAATGCTG	60.1	
*myo15a*	Forward	CATGGCACCCAGGAGATGATCTTG	59.7	136
	Reverse	CACGCTTGGCATTGTAGGCATTG	59.4	
*vwf*	Forward	CCACAAGGTCATTTCTCCAGCCAC	60.1	109
	Reverse	GGTCCGACAGAGGTGAGCATAAG	59.1	
*hprt*	Forward	AGTCCCAGCGTCGTGATTAGTGATG	60.4	140
	Reverse	CGAGCAAGTCTTTCAGTCCTGTCCA	60.5	

#### Real-time qPCR and analysis of expression data

qPCR was performed using Power SYBR® Green PCR Master Mix (Applied Biosystems, Darmstadt, Germany) on an ABI Prism 7300 Real Time PCR System (Applied Biosystems). Due to the large number of samples, qPCRs were performed on two 96-well plates for each target gene. For comparability, the normothermic ZT16 group was applied to all plates as inter-plate calibrator. *Hprt* was selected as reference gene, based on the stability of expression values across all samples. All samples were run in triplicates (for 5 biological replicates per group), using 1 μl cDNA as template in a reaction volume of 20 μl, and a series of six 10-fold dilutions of specific standard plasmids were used to generate the standard curve to calculate qPCR efficiencies. Additionally, a no-template control was included on each plate in duplicates for each target gene. Quantification was performed with the following cycling parameters for 40 cycles: 50°C 2 min; 95°C 10 min; 95°C 15 s; 60°C 15 s; 72°C 30 s. Amplification specificity was controlled by dissociation curve analysis referring to the qPCR run.

First evaluation of qPCR results was carried out using the 7300 System Software v1.4.0 (ABI Prism, Applied Biosystems) and subsequently exported to Microsoft Excel 2010 to identify differences in expression levels using the ΔΔCT method. All statistical testings and figures were done with SigmaPlot 12.5 (Systat Software Inc.). All results were statistically analyzed by two-way ANOVA with time of day (Zeitgebertime) and metabolic state (torpid/normothermic) as factors, followed by Tukey's test for pairwise comparison of relative expression levels between torpid and normothermic hamsters and within the torpid and normothermic groups.

## Results

### Transcriptomic expression analysis in the hypothalamus of *P. sungorus* during torpor entrance

We identified a total number of 27,830 transcripts with 284 transcripts being differentially expressed in hamsters during torpor entrance as compared to ZT matched normothermic hamsters (Table 2). A total of 181 transcripts were significantly upregulated whereas 103 transcripts were significantly downregulated. All transcripts identified had an RPKM-value ≥ 0.1 and a FDR *p* ≤ 0.05.

**Table 2:** **Overview of Illumina sequencing data and transcriptomic expression analysis**.

		**Up regulated**	**Down regulated**
Identified genes	27,830		
Differentially expressed genes	284	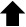 181	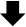 103

### Functional classification of differentially expressed genes

Differentially expressed transcripts during torpor entrance were classified according to gene ontology categories. It has to be noted, that some genes were included in more than one category.

**Figure 2:**
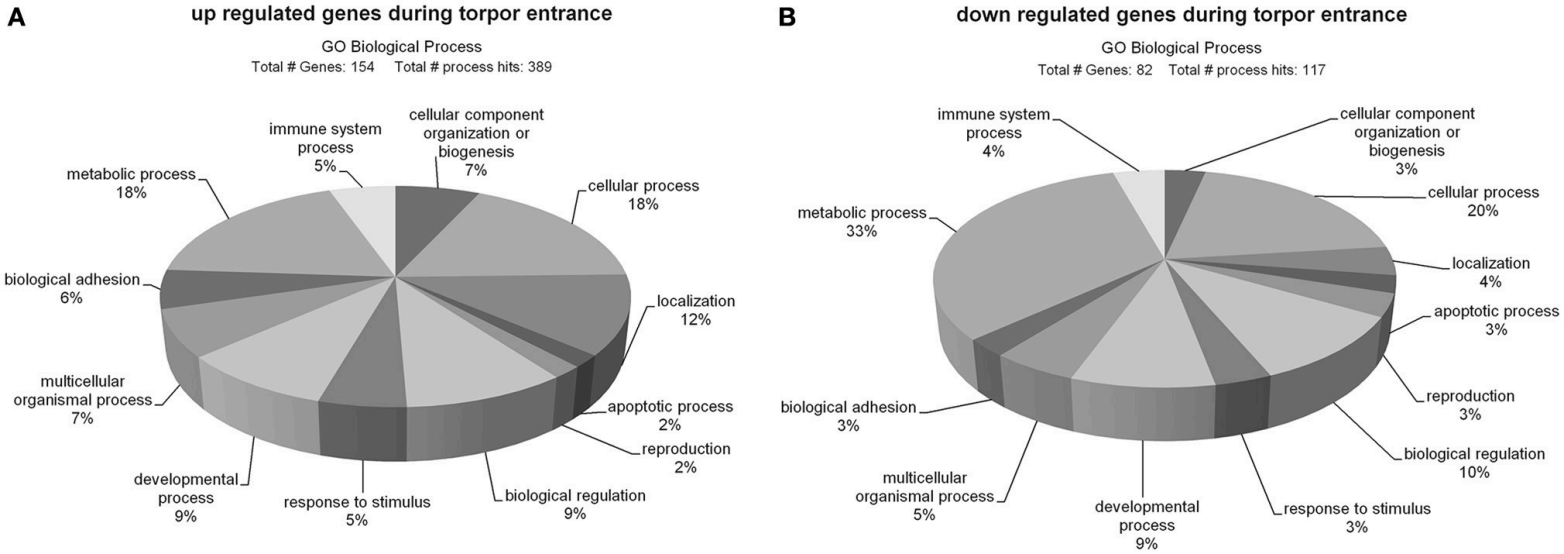
**Ontology of genes up- (A)** and down regulated **(B)** during torpor entrance in the hypothalamus. Sequences were classified according to http://pantherdb.org and assigned into biological process hits. Some genes were assigned to more than one category.

Of the 181 up regulated genes 154 could be classified and assigned to 389 biological processes (Figure 2A). The majority of up regulated genes are involved in metabolic (18%) and cellular processes (18%), followed by localization (12%), biological regulation (9%), developmental processes (9%), cellular component organization or biogenesis (7%), multicellular organismal processes (7%), biological adhesion (6%), immune system processes (5%), response to stimulus (5%), apoptotic processes (2%), and reproduction (2%).

A total number of 82 out of the 103 down regulated genes could be classified and assigned to 117 biological process hits (Figure 2B). The majority of down regulated genes was assigned to metabolic processes (33%), followed by cellular processes (20%), biological regulation (10%), developmental processes (9%), multicellular organismal processes (5%), localization (4%), immune system processes (4%), cellular component organization or biogenesis (3%), reproduction (3%), response to stimulus (3%), apoptotic processes (3%), and biological adhesion (3%).

The PANTHER overrepresentation test showed significant enrichments of the GO-slim terms only for the up regulated group of genes, comprising “transmembrane transporter activity” (9.64-fold, *p* = 0.034) in the domain molecular function and “extracellular matrix” (6.02-fold, *p* = 0.000397) in the domain cellular component.

### Analysis of most affected genes during torpor entrance

To determine the most affected genes during torpor entrance, we ranked the identified genes into the 20 most up- and 20 most down regulated genes, based on their fold changes.

Most up regulated genes (Table 3) showed fold changes in a range of 1.55–2.66. Within this group we found 8 genes coding for structure proteins (*LOC103164493, col20a1, myo15a, col17a1, micalcl, dnah2, col18a1, col5a3*), 4 involved in transporter function (*abca6, atp2a1, kcnh3, atp1a4*), 2 with signaling function (*OR2K2, LOC100773864*) and one gene each involved in stress defense (*klk8*), coagulation (*vwf*) and cell death (*steap3*). Three genes have so far unknown function (*LOC103160902_1, LOC100766933, catip*).

**Table 3:** **Most up regulated genes in the hypothalamus during torpor entrance**.

**Function**	**Gene**	**Gene symbol**	**Fold change**
Structure	Collagen alpha-1(XXIV) chain-like	*LOC103164493*	2.03
	Collagen, type XX, alpha 1	*col20a1*	1.97
	Myosin XVA	*myo15a*	1.93
	Collagen, type XVII, alpha 1	*col17a1*	1.77
	MICAL C-terminal like	*micalcl*	1.77
	Dynein, axonemal, heavy chain 2	*dnah2*	1.67
	Collagen, type XVIII, alpha 1	*col18a1*	1.62
	Collagen, type V, alpha 3	*col5a3*	1.55
Transporter	ATP-binding cassette, sub-family A (ABC1), member 6	*abca6*	2.07
	ATPase, Ca^++^ transporting, cardiac muscle, fast twitch 1	*atp2a1*	1.9
	Potassium voltage-gated channel, subfamily H, member 3	*kcnh3*	1.6
	ATPase, Na^+^/K^+^ transporting, alpha 4 polypeptide	*atp1a4*	1.58
Signaling	Olfactory receptor, family 2, subfamily K, member 2	*OR2K2*	2.66
	Cyclin-dependent kinase 11B-like	*LOC100773864*	1.67
Stress defense	Kallikrein-related peptidase 8	*klk8*	1.87
Coagulation	von Willebrand factor	*vwf*	1.59
Cell death	STEAP family member 3, metalloreductase	*steap3*	1.69
Unknown	EF-hand calcium-binding domain-containing protein 8	*LOC103160902_1*	1.94
	Protein ARMCX6-like	*LOC100766933*	1.73
	Ciliogenesis associated TTC17 interacting protein	*catip*	1.63

Fold changes of the most down regulated genes (Table 4) ranged between −1.5 and −4.0. This group contained 7 genes coding for transcription factors (*stk31, LOC100768314, LOC102638674, LOC100756005, LOC102632383, LOC102642077, smim11*), 4 with enzymatic activity (*top2a, clk1, coq3, LOC100772408_2*), 2 with transporter functions (*slc47a1, nipsnap3b*), one gene each involved in cellular structure (*cornifin-A*), signaling (*psmc3ip*), rRNA maturation (*rrp15*) and ORF (*swt1*) and 3 genes with so far unknown function (*LOC100754037, LOC103159055, LOC100753290*).

**Table 4:** **Most down regulated genes in the hypothalamus during torpor entrance**.

**Function**	**Gene**	**Gene symbol**	**Fold change**
Trancription factor	Serine/Threonine kinase 31	*stk31*	−2.69
	Zinc finger protein 93-like	*LOC100768314*	−1.69
	Zinc finger protein 26-like	*LOC102638674*	−1.62
	Zinc finger protein 420-like	*LOC100756005*	−1.61
	Zinc finger protein 431-like	*LOC102632383*	−1.56
	Zinc finger protein 431-like	*LOC102642077*	−1.55
	Small integral membrane protein 11	*smim11*	−1.47
Enzyme	Topoisomerase (DNA) II alpha 170kDa	*top2a*	−2.08
	CDC-like kinase 1	*clk1*	−1.93
	Coenzyme Q3 methyltransferase	*coq3*	−1.51
	2-hydroxyacylsphingosine 1-beta-galactosyltransferase	*LOC100772408_2*	
Transporter	Solute carrier family 47 (multidrug and toxin extrusion), member 1	*slc47a1*	−1.87
	Nipsnap homolog 3B	*nipsnap3b*	−1.48
Structure	SMALL PROLINE-RICH PROTEIN 1A	*cornifin-A*	−4.04
Signaling	PSMC3 interacting protein	*psmc3ip*	−1.71
rRNA maturation	Ribosomal RNA processing 15 homolog	*rrp15*	−1.61
ORF	SWT1 RNA endoribonuclease homolog	*swt1*	−1.56
Unknown	Chromosome unknown open reading frame, human C5orf46	*LOC100754037*	−2.99
	Uncharacterized LOC103159055	*LOC103159055*	−2.87
	Chromosome unknown open reading frame, human C5orf63	*LOC100753290*	−1.51

### Verification of most up regulated gene expression by qPCR

**Figure 3:**
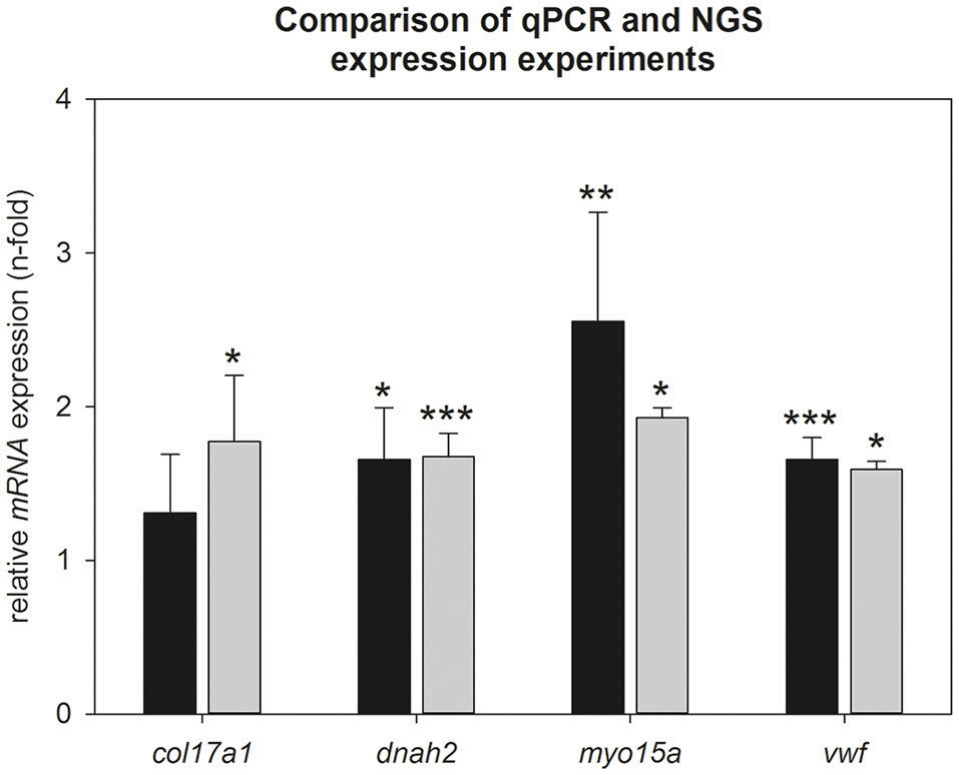
**Comparison of NGS- and qPCR data during torpor entrance**. Expression changes were calculated by comparison of torpid animals at ZT1 to normothermic animals at ZT1 in both experiments. Gray bars represent torpid animals at ZT1(±SEM) analyzed by NGS and black bars represent torpid animals at ZT1(±SEM) analyzed by qPCR. Significant differences to their relative control groups are marked with ^*^p < 0.05, ^**^p < 0.01, ^***^p < 0.001.

To verify the Illumina results, we calculated hypothalamic relative mRNA expression of *col17a1, dnah2, myo15a*, and *vwf* during torpor entrance by qPCR (Figure 3). Up regulation could be confirmed for *dnah2* (qPCR: 1.6-fold, *p* = 0.016; Illumina: 1.7-fold, *p* < 0.001), *myo15a* (qPCR: 2.5-fold, *p* = 0.005; Illumina: 1.9-fold, *p* = 0.035) and *vwf* (qPCR: 1.6-fold, *p* < 0.001; Illumina: 1.6-fold; *p* = 0.046). Up regulation of *col17a1* did not reach significance in the qPCR analysis (qPCR: 1.2-fold, *p* = 0.462; Illumina: 1.8-fold, *p* = 0.027). Also the other collagens identified by Illumina did not reach significance by qPCR (*col5a3*: 1.3-fold, *p* = 0.550; *col18a1*: 1.2-fold, *p* = 0.450; *col20a1*: 2.0-fold, *p* = 0.361; *LOC103164493*: 1.4-fold, *p* = 0.563) (data not shown).

### Relative gene expression patterns over the circadian cycle in torpid and normothermic hamsters

**Figure 4:**
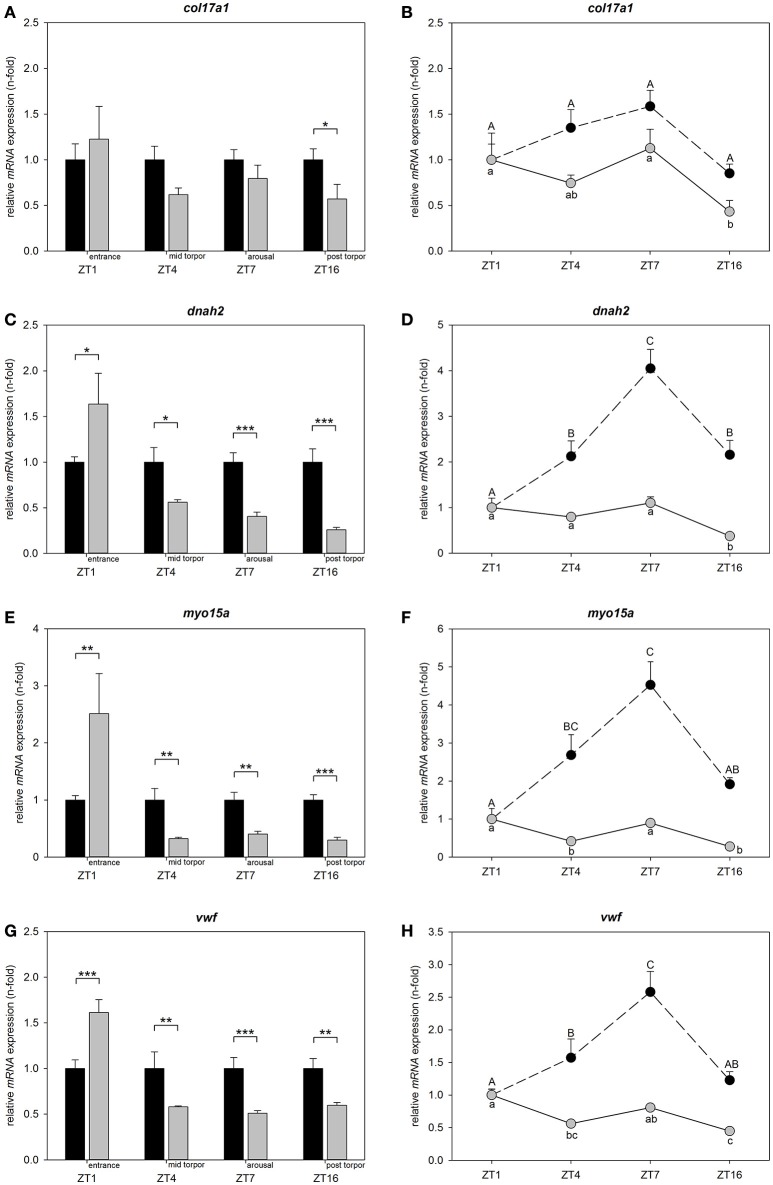
**Circadian regulation of *col17a1*, *dnah2*, *myo15a*, and *vwf* in torpid and normothermic Djungarian hamsters**. Bar graphs on the left side show differences in *mRNA* expression of *col17a1* **(A)**, *dnah2* **(C)**, *myo15a* **(E)**, and *vwf* **(G)** in torpid animals (gray bars, ±SEM) relative to normothermic control group at same ZT (black bars, ±SEM). Significant differences are marked with ^*^p < 0.05, ^**^p < 0.01, and ^***^p < 0.001. Line graphs on the right side show relative differences in *mRNA* expression of *col17a1* **(B)**, *dnah2* **(D)**, *myo15a* **(F)**, and *vwf* **(H)** over the course of a day within normothermic animals relative to normothermic ZT1 group marked with upper case (black circles, ±SEM) and within torpid animals relative to torpid ZT1 group marked with lower case (gray circles, ±SEM). Data points with different characters are significantly different (*p* < 0.05).

To determine, whether differential candidate gene expression is restricted to torpor entrance and to assess circadian regulation, we investigated relative mRNA expression at ZT1, ZT4, ZT7, and ZT16 in animals undergoing torpor and animals remaining normothermic. Differences within each investigated time point are shown relative to normothermic control group at same ZT respectively (Figures 4A,C,E,G). Circadian variations for normothermic animals are shown relative to the normothermic ZT1 group. Circadian variations for torpid animals are presented relative to torpor ZT1 group (Figures 4B,D,F,H).

There was no effect of time of day on *col17a1* mRNA levels for normothermic animals, but there was an effect of time of day for torpid animals (two-way ANOVA: *p* < 0.001). *Col17a1* mRNA expression was reduced in the post-torpor group (ZT16) as compared to torpor entrance (ZT1, Tukey's test: *p* = 0.004) and to arousal (ZT7, Tukey's test: *p* = 0.002) (Figure 4B).

There were no significant changes in mRNA expression during torpor entrance (ZT1), mid torpor (ZT4) or arousal (ZT7) relative to normothermic control groups at the same ZTs. Post-torpor (ZT16), mRNA expression was 0.57-fold down regulated (Tukey's test: *p* = 0.014) (Figure 4A).

There was an effect of time of day on *dnah2* mRNA levels both, in normothermic and torpid animals (two-way ANOVA: *p* < 0.001). Normothermic animals showed lowest mRNA expression at ZT1 (Tukey's test: ZT1 vs. ZT4 *p* = 0.006, ZT1 vs. ZT7 *p* < 0.001, ZT1 vs. ZT16 *p* = 0.004) that increased at ZT4, peaked at ZT7 (Tukey's test: ZT7 vs. ZT4 *p* = 0.011) and decreased again at ZT16 (Tukey's test: ZT16 vs. ZT7 *p* = 0.015) (Figure 4D). Over the investigated torpor stages (ZT1, 4, 7) no significant changes were found, but post-torpor (ZT16) mRNA expression was 0.38-fold down regulated as compared to torpor entrance (ZT1, Tukey's test *p* < 0.001), mid torpor (ZT4, Tukey's test *p* = 0.003) and arousal (ZT7, Tukey's test *p* < 0.001) (Figure 4D).

Relative to their normothermic control groups, *dnah2* expression was 1.64-fold up regulated during torpor entrance (ZT1, Tukey's test *p* = 0.016), 0.56-fold down regulated during mid-torpor (ZT4, Tukey's test *p* = 0.014), 0.40-fold down regulated during arousal (ZT7, Tukey's test *p* < 0.001) and 0.26-fold down regulated post-torpor (ZT16, Tukey's test *p* < 0.001) (Figure 4C).

There was an effect of time of day on *myo15a* mRNA levels both, in normothermic and torpid animals (two-way ANOVA: *p* < 0.001). Normothermic animals showed low mRNA expression at ZT1 that increased at ZT4 (Tukey's test *p* = 0.019) and ZT7 (Tukey's test *p* < 0.001), before decreasing again at ZT16 (Tukey's test: ZT16 vs. ZT7 *p* = 0.030). Torpid animals showed highest mRNA expression during torpor entrance (ZT1) differing significantly from mid torpor (ZT4, Tukey's test *p* = 0.010) and post-torpor (ZT16, Tukey's test *p* < 0.001). mRNA expression at mid torpor (ZT4) was also down regulated as compared to arousal (ZT7, Tukey's test *p* = 0.047) and mRNA expression during arousal (ZT7) was up regulated relative to post-torpor (ZT16, Tukey's test *p* < 0.001) (Figure 4F).

*Myo15a* expression was 2.51-fold up regulated during torpor entrance (ZT1, Tukey's test *p* = 0.005), 0.32-fold down regulated at mid torpor (ZT4, Tukey's test *p* = 0.002), 0.40-fold down regulated during arousal (ZT7, Tukey's test *p* = 0.006) and 0.29-fold down regulated in post-torpor group (ZT16, Tukey's test *p* < 0.001) (Figure 4E) as compared to the normothermic control groups.

There was an effect of time of day on *vwf* mRNA levels both, in normothermic and torpid animals (two-way ANOVA: *p* < 0.001). Normothermic animals showed low mRNA expression at ZT1 that increased at ZT4 (Tukey's test *p* = 0.038) and further at ZT7 (Tukey's test: ZT1 vs. ZT7 *p* < 0.001, ZT4 vs. ZT7 *p* = 0.017), before decreasing at ZT16 (Tukey's test: ZT7 vs. ZT16 *p* < 0.001). The mRNA expression in torpid animals was significantly up regulated during torpor entrance (ZT1) as compared to mid torpor (ZT4, Tukey's test *p* = 0.002) and post-torpor (ZT16, Tukey's test *p* < 0.001) and during arousal (ZT7) compared to post-torpor (ZT16, Tukey's test *p* = 0.002) (Figure 4H).

*Vwf* expression was 1.61-fold up regulated during torpor entrance (ZT1, Tukey's test *p* < 0.001), 0.58-fold down regulated during mid-torpor (ZT4, Tukey's test *p* = 0.004), 0.51-fold down regulated during arousal (ZT7, Tukey's test *p* < 0.001) and 0.60-fold down regulated in post-torpor group (ZT16, Tukey's test *p* = 0.003) (Figure 4G) as compared to the normothermic control groups.

## Discussion

Our data show 284 differentially expressed genes out of 27,830 identified genes in the hypothalamus of *P. sungorus* during entrance into the torpid state, implying that just a small set of genes is affected by the metabolic depression initiating torpor entrance. These results are in accordance with previous studies showing that transcript levels of most genes are unaffected during torpor (Storey and Storey, 2004). In accordance with the fact that daily torpor is a state of extreme metabolic adjustment, the majority of differentially regulated genes was found in cellular and metabolic processes for both, up and down regulated genes.

The majority of the top 20 down regulated genes were transcription factors, which could be responsible for a delay or suppression of mRNA transcription during the torpid state. It has been shown before, that transcriptional initiation as well as elongation rates are reduced during hibernation in golden-mantled ground squirrels (van Breukelen and Martin, 2002). Also in *P. sungorus* metabolic depression is associated with reduced transcriptional initiation (Berriel Diaz et al., 2004). This may contribute to the generally suppressed protein synthesis during torpor that has been demonstrated in various tissues from different species (Gulevsky et al., 1992; Frerichs et al., 1998; Hittel and Storey, 2002).

Within the top 20 up regulated group our data show a remarkable number of genes coding for structure proteins. Except for the up regulation in collagen genes we were able to verify these results by qPCR for *dnah2, myo15a* and the procoagulation factor *vwf*.

Collagens are extracellular matrix structural components, which are involved in neuronal development of the brain. Collagens play a role in axonal guidance, synaptogenesis and establishment of brain architecture (Chernousov et al., 2006; Fox, 2008; Hubert et al., 2009). A study of Schwartz et al. (2013) identified an up regulation of several collagen genes in the cerebral cortex, but not in the hypothalamus, of thirteen-lined ground squirrels during deep hibernation and interbout arousals, indicating synaptic plasticity during hibernation (Schwartz et al., 2013). Although we obtained a significant up regulation in mRNA expression of five collagen genes during torpor entrance by NGS and a significant enrichment of extracellular matrix components in up regulated gens, we were not able to verify these results by qPCR. There was only a trend of increased *col17a1* during torpor entrance as well as slightly lower mRNA levels during all other torpor stages and no diurnal changes could be detected in normothermic animals. Investigation of all other collagens identified in the 20 up regulated group showed a similar picture with a slight up regulation at torpor entrance and trend to lower mRNA levels during the other torpor stages that did not reach significance (data not shown). There was a high variability in the mRNA expression levels of qPCR samples, especially at torpor entrance, which might have caused the non-significant result. Different groups of animals were used for NGS and qPCR study and data might reflect inter-individual differences. A larger sample size might help to resolve expression patterns in collagen genes more precisely. Hence, whether collagens are involved in synaptic remodeling and plasticity during torpid states remains to be revealed.

Elevated expression of *dnah2*- and *myo15a* mRNA during torpor entrance could be identified by both, NGS and qPCR approach. Myosin and dynein are structural components of cytoskeleton and represent two out of three superfamilies of molecular motor proteins in neurons. They are able to transport biomolecules, such as vesicles, protein complexes and mRNAs in axons, dendrites and pre- and post-synaptic regions. Intracellular transport is necessary for neuronal morphogenesis, function and survival (Hirokawa et al., 1998, 2010; Vale, 2003). During deep hibernation, elevated mRNA levels of three different myosin types and one dynein have been detected in the cerebral cortex of *S. tridecemlineatus*, indicating dynamic structural changes (Schwartz et al., 2013).

In our study, hamsters showed elevated *dnah2* and *myo15a* expression only during torpor entrance (ZT1), whereas mRNA expression was reduced at all other investigated torpor states (mid torpor, arousal, post-torpor) compared to normothermic animals. The higher expression of *dnah2* and *myo15a* during torpor entrance could be important to ensure maintenance of synaptic transmission and neuron survival during torpor by an elevated transport of biomolecules. It might also be possible that higher mRNA amounts are produced at the beginning and stored during the torpid state to provide transcripts for a fast utilization of these molecular motors during arousal. However, we think this possibility is unlikely because mRNA levels are already declining during mid-torpor (ZT4).

In normothermic animals *dnah2* as well as *myo15a* show a diurnal regulation in its mRNA expression with a peak at ZT7 in normothermic animals. This might suggest a higher demand of these motor proteins during the hamster's naturally active phase.

Taken together changes in structural protein shows evidence for plasticity in the hypothalamus of torpid hamsters and thereby confirm studies in deep hibernation that have proposed plastic changes in the brain before.

In addition to structure gene expression changes, we chose to investigate *vwf* in more detail, because of its function in blood clotting. In torpid animals the reduced heart rate, ventilation and T_b_ results in a decreased blood flow that increases relatively fast to its euthermic flow rate during arousal. In contrast to all other mammalian species, torpor expressing mammals are able to survive these periods of low blood flow and consequent reperfusion without apparent formations of deep vein thrombi, stroke or pulmonary embolism (Lyman and O'Brien, 1961; Frerichs et al., 1994).

vWF is a major factor involved in platelet adhesion and thrombus formation (Denis and Wagner, 2007). Higher vWF levels increase the risk for thrombosis and embolism whereas deficiency in vWF activity leads to the human bleeding disorder von Willebrand's disease (Sadler, 1998, 2005). Moreover, Zhao et al. (2009) identified vWF as an important protein regulating the occurrence of cerebral ischemia and showed that a lack of vWF is able to reduce infarct volume (Zhao et al., 2009). Based on this knowledge, a reduced level of vWF would be expected during the torpid state to prevent blood clotting during periods of low blood flow. Indeed, in plasma samples of hibernating thirteen-lined ground squirrels vWF collagen binding is 10-fold decreased and in lung tissues *vwf* mRNA expression is 3-fold down regulated during torpor (Cooper et al., 2016). Unexpectedly, our NGS and qPCR data show an elevated level of *vwf* mRNA during torpor entrance in the hypothalamus. The elevated level of *vwf* mRNA might either not directly translate into protein variation or alternatively translate into protein without damaging effects, namely inactive vWF. vWF is a large multimeric glycoprotein which can be cleaved in smaller multimers by ADAMTS13, a zinc-containing metalloprotease enzyme. These smaller multimers of vWF have a strongly decreased activity resulting in a reduced platelet adhesion and aggregation (Chauhan et al., 2006; Zhao et al., 2009). In this case, no damage of brain structures would be expected even when higher *vwf* levels are present. Moreover, apart from the up regulation during torpor entrance, *vwf* expression was lower in torpid animals at all other investigated states, supporting the hypothesis of low *vwf* levels facilitating blood flow during the torpid state. Diurnal changes of *vwf* could be detected in either group. Normothermic animals displayed highest *vwf* level at ZT7, suggesting a higher demand of *vwf* at the beginning of the active time. In torpid animals *vwf* level is lowest at mid torpor (ZT4) and post-torpor (ZT16). Taken together, our data provide evidence for readjustment of blood clotting during different torpor stages as well as times of day.

In general, the diurnal mRNA expression of all investigated genes of this study is less pronounced in torpid animals, which is likely to be caused by the suppression of transcription and translation during torpor. The transcriptional depression during torpor has been shown to result from both, down regulated transcriptional initiation and suppressed elongation (van Breukelen and Martin, 2002; Berriel Diaz et al., 2004). Low T_b_ during torpor affects biochemical process, leading to a decline in gene expression caused by the temperature sensitivity of transcriptional elongation (van Breukelen and Martin, 2002; Berriel Diaz et al., 2004).

The NGS technology allows a whole transcriptome survey of gene expression changes and our analysis provide an overview of gene expression changes during torpor initiation in *P. sungorus* for the first time.

Although we could not determine signaling pathways regulating torpor initiation with this approach, we identified molecular adaptions in the hypothalamus of *P. sungorus* initiated during the early state of torpor. Our data provide evidence for synaptic remodeling and plasticity, an elevated transport of biomolecules and readjustment of coagulation. Comparable gene expression changes have already been found in deep hibernators. This would support the hypothesis that daily torpor and hibernation are similar physiological states only differing in amplitude and duration. Interestingly, the molecular changes already occur within the short time span of daily torpor. These adaptations may, just like in deep hibernation, help the brain cells to better survive or reduce cell damages during the extreme physiological conditions in the torpid state. In the future, precise anatomical investigation of identified genes is necessary to eventually gain insights into their functions.

## Author contributions

CC, AF, and AH designed experiments, CC and JK performed experiments. CC, JK, AF, and AH analyzed and interpreted the data. CC and AH drafted the manuscript which was critically revised by JK and AF.

## Funding

This work was funded by the German Research Foundation (DFG, Emmy-Noether HE6383 to AH).

### Conflict of interest statement

The authors declare that the research was conducted in the absence of any commercial or financial relationships that could be construed as a potential conflict of interest.

## Abbreviations

*col17a1*: collagen, type XVII, alpha 1
*dnah2*: dynein, axonemal, heavy chain 2
*myo15a*: myosin XVA
NGS: next generation sequencing
qPCR: quantitative real-time PCR
T_a_: ambient temperature
T_b_: body temperature
*vwf*: von Willebrand factor
ZT: Zeitgebertime.
